# Acceptance of virtual patients as a continuous professional development approach among practicing nurses in primary health care settings in a low-income country: a quasi-experimental posttest setup design

**DOI:** 10.1186/s12912-024-02000-0

**Published:** 2024-05-16

**Authors:** Gerard Nyiringango, Uno Fors, David K. Tumusiime, Elenita Forsberg

**Affiliations:** 1 Department of Computer and Systems Sciences, Stockholm University, Borgarfjordsgatan 12, PO Box 7003, Stockholm, SE-164 07 Sweden; 2 School of Nursing and Midwifery, College of Medicine and Health Sciences, University of Rwanda, P.O.Box 3286, Kigali, Rwanda; 3 School of Health Sciences, College of Medicine and Health Sciences, University of Rwanda, Kigali, Rwanda; 4 School of Health and Welfare, Halmstad University, Halmstad, Sweden

**Keywords:** Virtual patient, Healthcare education, Continuous professional development, Continuing nursing education, Nurses, Primary healthcare settings, Low-income countries

## Abstract

**Background:**

Virtual patients are an educational technological approach used in healthcare education. Its distinctive features have rendered virtual patient technology appealing for the training of medical and healthcare students, particularly in the enhancement of clinical reasoning. Virtual patients are less often applied for continuous professional development for practicing healthcare providers, and there is a scarcity of studies exploring this possibility. This study aimed to assess the acceptability of nurses for using virtual patients as a continuous professional development approach.

**Method:**

The study used a quasi-experimental posttest setup design. The study was conducted in ten primary healthcare settings in Rwanda. Among 76 nurses who consented to participate in the study, 56 completed the intervention and responded to the study questionnaire. Following a one-week program of continuous professional development on four non-communicable diseases, the study used a self-administered questionnaire based on the Technology Acceptance Model 3 to collect data. Descriptive analysis served as the primary method for analyzing participants’ responses. The study also used a correlation test to assess the relationship of variables.

**Results:**

Across all items in the questionnaire, the median response tended towards either agree or strongly agree, with only a minority number of participants expressing strong disagreement, disagreement, or neutrality. The results indicated a significant positive correlation between perceived usefulness and behavior intention (*p* < 0.001).

**Conclusion:**

The findings indicate an acceptability and behavioral intention of adopting virtual patients as an alternative continuous professional development approach among nurses working at health centers in Rwanda or other locations with similar contexts.

**What is already known**.

- The use of VPs is a well-established technology in healthcare education institutions for teaching and learning purposes.
- The VP learning approach enhances clinical reasoning, communication, and ethics understanding among healthcare students.
- There is a lack of studies exploring the possibility of utilizing VP technology for CPD of in-service healthcare providers.

**What this paper adds**.

- This study indicated the potential for utilizing a VP approach in the CPD of in-service nurses.
- Nurses view VPs as useful and exhibit a behavioral intention to use them as an alternative approach for CPD.

## Background

The contemporary use of information technology in education has enabled various development of approaches that facilitate teaching and learning for both pre- and in-service education. Virtual patients (VPs) are one of these approaches and many higher learning institutions have started to use it as part of the curriculum for medical and healthcare students. “VPs are interactive computer simulations of real-life clinical scenarios for the purpose of healthcare and medical training, education, or assessment” [1]. The nature of interaction when using VPs varies based on the specific learning objectives and the chosen VP system. Generally, however, VP enables the learner to “ask” questions (type or select a pre-formulated question) about a clinical scenario and the VP system provides a response a patient would do [2]. Such clinical scenarios displayed on a computer or tablet screen enable users to perform comprehensive tasks including taking a patient’s history, conducting a physical examination, ordering laboratory or imaging tests, formulating a diagnosis, and subsequently devising a management plan, plus receiving feedback on actions taken.

These distinctive features of VP technology have made it appealing for the training of medical and healthcare students, especially in the context of developing clinical reasoning skills [3–5]. Moreover, studies indicate that learners in pre-service education appreciate and accept the use of VPs for learning [6, 7]. However, while, VPs have the potential of being used for in-service training, there is limited evidence that it can be accepted by in-service healthcare providers for continuous professional development (CPD) In this study, the researchers conducted a one-week training for nurses working at the health center level in Rwanda and thereafter, assessed the nurses’ acceptability in embracing VPs as a tool for CPD. The study used the technology acceptance model [8, 9] to describe the acceptability of nurses regarding the use of VPs for CPD if it is implemented as a CPD approach.

### Context of the study

The study was carried out in Rwanda, a lower-income country in East Africa with a population of 13 million. Rwanda employs a pyramidal health system, with community health workers and dispensaries forming the base, followed by health centers, district hospitals, provincial referral hospitals, and ultimately tertiary hospitals at the apex. Many countries in sub-Saharan Africa adhere to this health system structure. The health center level, responsible for delivering healthcare services to over 80% of Rwandans, is staffed by nurses who receive their training in nursing during their pre-service education [10]. Already when beginning work at the health centers, nurses are allowed to start working in consultation rooms and immediately start deciding on patients’ cases by either transferring the case to the next health system level or managing the patient at the health center, meaning a work task that often is performed by physicians in countries with higher income and better access to physicians. Thus, these nurses have a very important role and are making decisions that influence both patient’s health and the use of scarce resources like hospital visits, physicians, intake to local wards at the health center, and/or medications. In this context, a nurse who did not train to primarily master the medical workflows, and who has not had the opportunity to work alongside experienced individuals at the bedside may lack certain skills in investigating clinical cases and making appropriate decisions about them. On one hand, the Ministry of Health deliberately employs information workflows about specific types of diseases, enabling nurses at this level to make informed decisions, as well as offering both face-to-face and online training sessions to enhance decision-making skills. On the other hand, however, inconclusive evidence reveals that VPs have the potential to expose nurses at the level of health centers to numerous disease cases that were developed by experts for learning purposes which may contribute to their decision-making abilities [11]. In this regard, the VP can serve as a learning approach that connects senior professionals from higher institutional levels to junior ones at health centers. In line with this, Mugo et al., [12] argue that teaching and learning technology should be preceded by an assessment of user acceptance of that technology. Thus, there is a need to understand the acceptance behavior of nurses toward the use of VPs for CPD. This study aimed to assess the acceptability of nurses for using VPs as a CPD approach.

### Theoretical foundation

The study used the technology acceptance model to assess nurses’ acceptance of VPs for CPD. Davis [13], defines acceptance as the motivation or likelihood of using the alternative proposed system. The technology acceptance model deposits that the inclination behavior towards accepting a technology can be measured using the user’s attitude towards the technology [13]. Consequently, authors who contributed to the development of the technology acceptance model (TAM) have proposed various variables that can used to assess the user acceptability of a technology or an alternative system [8, 9, 12, 13]. As TAM evolved, additional variables were introduced or existing ones were modified. In this study, we adopted Technology Acceptance Model 3 (TAM3), as proposed by Venkatesh and Bala [8]. TAM3 has the variables of perceived usefulness, perceived ease to use, job relevancy (training relevancy), results demonstrability, perceived external control, and behavior intention [8]. The overall outcome variable in this study is behavior intention about using VPs for CPD. The technology acceptance model deposits that perceived usefulness and ease of use are major two determinants that predict the behavior intention of using a technology. Davis [14] defines perceived usefulness as “the degree to which a person believes that using a particular system would enhance his or her job performance” (p. 320). Davis [14] also defines ease to use as “the degree to which a person believes that using a particular system would be free of effort” (p.320).

The variables of results demonstrability, job relevance, and external control directly influence the perceived usefulness [8, 9]. The technology acceptance model defines these variables as follows: results demonstrability is the extent to which an individual believes that the results of using technology are tangible, observable, and communicable [15] Job relevance is the degree to which the individual believes technology applies to her or his job [9], and external control is the degree to which the individual believes the organization’s resources and support are available [8].

### Purpose

The purpose of this study was to assess the acceptability of using VP cases as a CPD approach among nurses at health centers.

### Research question

The main research question formulated was: What is the level of acceptance among nurses working in primary healthcare settings regarding the use of VPs for CPD?

## Methods

### Research design

In this study, we used a quasi-experimental posttest setup design, utilizing a self-administered validated questionnaire to assess the acceptance of nurses toward the utilization of VPs for CPD. The research design focused on describing the nurses’ acceptability of using VPs as an approach for CPD, following a one-week training on non-communicable diseases using the VP approach.

### Sample size calculation, study setting and participants

To determine the appropriate sample size, we used G*power 3.1. The sample size determination took account the correlation test, which assessed the correlation among scales, with an alpha value of 0.05, a power level of 0.95, and an effect size of 0.5, guiding the calculation. According to the G*Power calculation, 32 participants were deemed necessary for this study. However, anticipating a notable attrition rate due to the study’s design, we aimed to recruit double the number of participants.The study was conducted at primary healthcare settings known as health centers in Rwanda. Rwanda has a total of 499 health centers, evenly distributed across all four provinces and Kigali city [16]. Serving as primary healthcare facility, health centers in Rwanda provides a range of services, including preventive, curative, and rehabilitative care, and approximately employ between 10 and 20 nurses. For participant selection, two health centers were randomly selected from each Province and Kigali city, totaling 10 health centers. At the health center, the inclusion criteria to participate in the study considered if a nurse works in a consultation room and can read and understand English. The requirement of understanding English was due to the reason that the VP cases applied in this study were available only in English (even if the VP system used may host cases in other languages). A nurse who verbalized to meet these two criteria was requested to participate in the study. The aim was to include between four and eight nurses from each health center. If a nurse selected did not meet the inclusion criteria or declined participation, they were randomly replaced by another nurse. In total 76 nurses agreed to participate in the study but only 56 completed the scheduled CPD of four VP cases about non-communicable diseases. These 56 nurses responded to self-administered questionnaires about VP acceptance as a CPD approach.

### Virtual case system and VP cases

The CPD program using VP cases, started when researchers adapted an existing system known as the virtual case system (VCS) to fit it into the existing context of health centers in Rwanda. The VCS was developed by Stockholm University in Sweden and is primarily designed to train healthcare students. As mentioned before, nurses in the consultation room in Rwanda, receive patients and undergo a medical model diagnosis process which enables them to decide whether to transfer the patient to the district hospital or manage the case at the health center. Based on this context, the authors designed VP cases about non-communicable diseases of hypertension, gastric cancer, depression, and prostate cancer, which were selected based on their importance and commonness. These VP cases were designed and divided into six cycles in alignment with an authentic patient consultation at a health center in Rwanda: patient information, history taking, physical examination or status, laboratory examination, decision about the patient’s case (assessment), and feedback. The decision about the patient’s case was structured in the form of questions where a nurse was requested to propose a diagnosis and manage the case. In this section of the assessment, the nurse could answer other questions like the risk factors, or complications. The feedback section indicates the response of the nurse and an expert’s suggestion about the case.

The four non-communicable VP cases drew inspiration from the existing cases documented in patients’ files at health centers. However, the research team removed all patient identifiers and incorporated additional information to align with the intended learning objectives. The team drafted VP cases and nurses with educational and practice backgrounds related to each disease case validated them through the following process:

1. One author (N.G.) collaborated with nurses at a health center not involved in the study to extract pertinent cases from authentic patient files.
2. The research team meticulously reviewed the case contents, making modifications to the specific learning outcomes.
3. The case underwent scrutiny by local nurse experts, who provided valuable comments and suggestions.
4. The research team diligently addressed the received comments, refining the cases as needed.
5. The adapted cases returned to the experts for additional inputs and comments.
6. The cases underwent a pilot phase involving three nurses working at a health center.

**Fig. 1:**
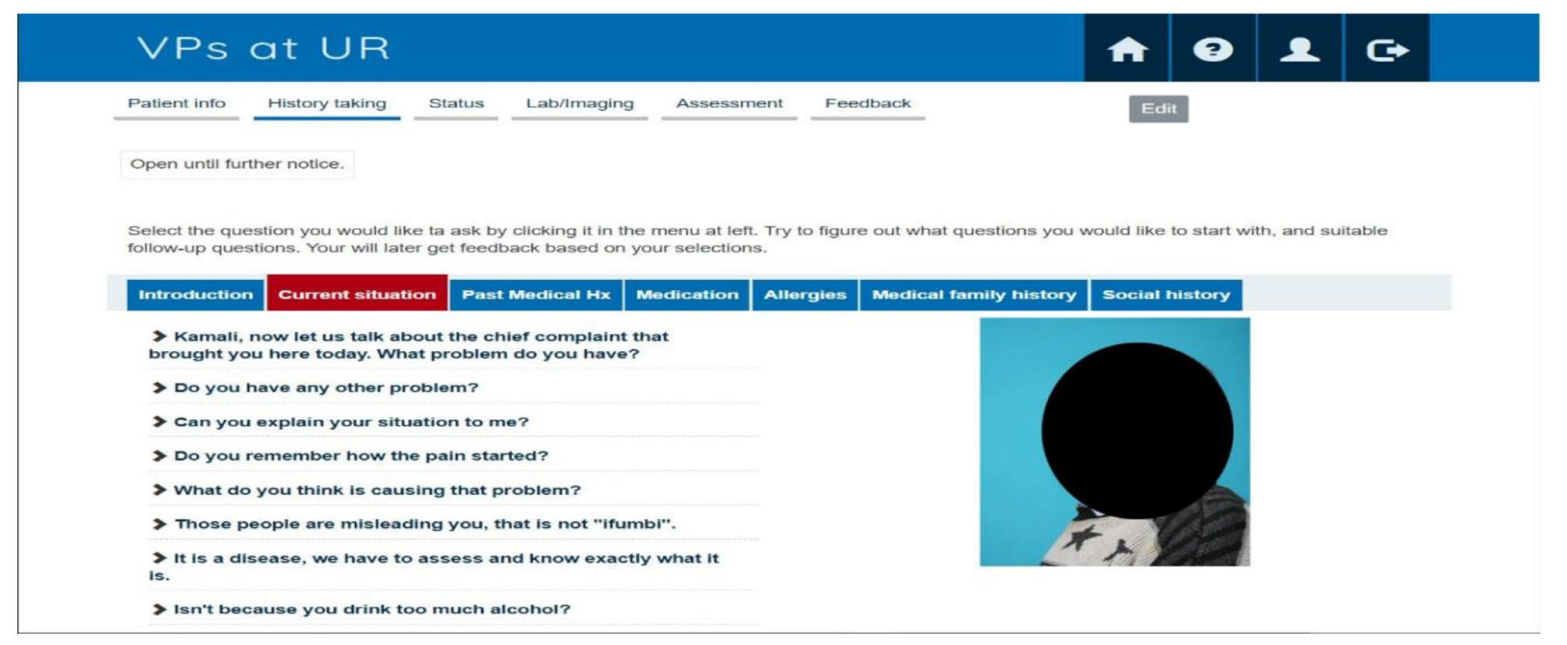
The screenshot image of history-taking

**Fig. 2:**
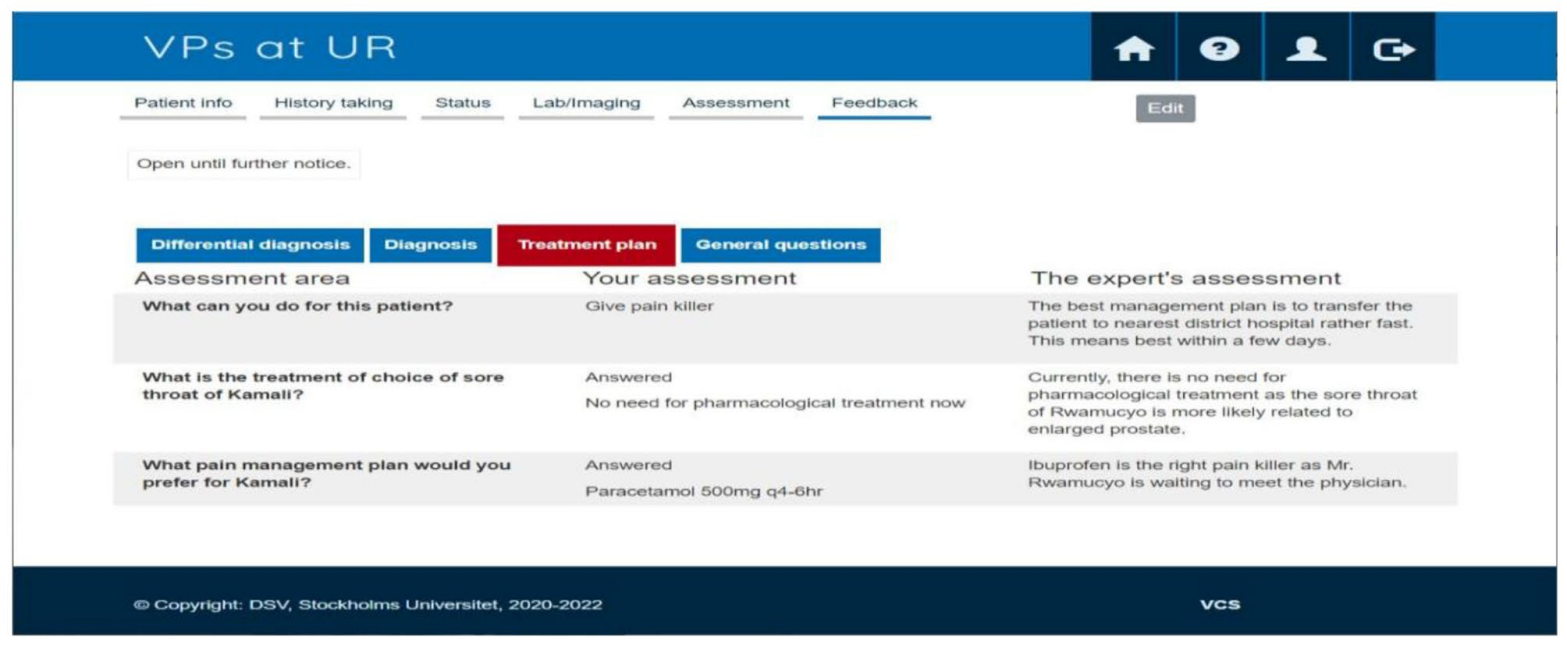
The screenshot image of the feedback feature

Finally, the research team and nurse experts who were involved in the validation process met in a workshop that approved all the cases. At this stage, all cases were entered into the VP system, making the completion of the meticulous development and validation process. Figures 1 and 2 show examples of VP case features.

Figure 1 is a screenshot illustrating certain questions in the history-taking process. When the learner clicks on each of these questions, a corresponding response pops up, prompting reflection on the suitability of the question to pursue further.

Figure 2 illustrates a selection of assessment questions, a participant’s- responses, and an expert’s recommendations.

### Tool for data collection

The study adopted the validated questionnaire-based technology acceptance model 3 [8] which encompasses numerous variables. However, for this study, we used variables of perceived usefulness, ease of use, results demonstrability, training relevance, perceived external control, and behavior intention. The original term for “training relevance” is “job relevance” [8]. However, in this study, we opted for “training relevance” as the focus is on acceptance of the training approach rather than the job itself. All the variables were measured using a Likert scale ranging from “strongly agree” (5), “agree” (4), “neutral” (3), “disagree” (2), to “strongly disagree” (1). The selection of a five-point Likert scale for this study was deliberate, aiming to offer respondents clear and distinct options for expressing their preferences. This scale choice aligns with the methodology used in technology acceptance model 3 [8]. The questionnaire was also piloted with three nurses working at health centers to validate its effectiveness. Furthermore, we assessed the questionnaire’s reliability using Cronbach’s Alpha, yielding an average Cronbach’s Alpha exceeding 0.7 for all items, indicating strong internal consistency.

### Data collection process

After randomly selecting ten health centers nationwide, two health centers from each province and Kigali city, we reached out to the respective heads of these health centers, seeking appointments for face-to-face meetings. In these meetings, we presented our ethical approvals and sought permission to contact potential participants within their facilities. Subsequently, we introduced our research idea during morning staff meetings at each health center, inviting nurses to participate in the study. Those who expressed willingness to participate and met inclusion criteria signed a consent form, and we mutually agreed on the commencement date of the study.

The study started by asking the nurses to work on four VP cases. Before starting, we provided a brief guidance session on navigating the VP cases. Following this introduction, we lent each participant a tablet equipped with internet access. according to the pilot study, each case lasted an average of 45 min. However, participants worked on these four VP cases for the whole week since they continued also doing other daily tasks.

Upon the completion of all VP cases, participants received a Google link containing a questionnaire on the acceptance of VP cases for CPD. The study intervention and data collection took one week at each health center, starting on 13th March 2023 and ending on 8th June 2023.

### Data analysis

The study used SPSS version 28 to describe the participants’ acceptance of utilizing VPs for CPD. While most previous studies utilizing the technology acceptance model focused on predicting behavior intentions, our approach delved into describing the variables that underlie behavioral intention. The nature of the study and the limited sample size of 56 constrained us from employing predictive statistics. In this study, behavior intention serves as the dependent variable, with perceived usefulness and perceived ease of use serving as independent variables that predict behavior intention. For the description of the study’s outcome variables, we presented the median, minimum, and maximum values, reflecting participants’ agreement with statements regarding the use of VPs in CPD. Additionally, we transformed these ordinal variables into scale variables by summing up values from 1 to 5, which permitted us to ascertain the correlational relationships among the variables. The study used a two-tailed approach and an alpha level of 0.05 to define statistical significance.

## Results

The results of the study are presented according to participants’ responses about their behavioral intention to accept VPs as an approach for CPD among nurses in health centers in Rwanda. The research included 56 nurses from 10 health centers situated in various locations across Rwanda. Table 1, provides an overview of the demographic characteristics of participants:

**Table 1:** Demographic characteristics of participants

Item		Frequency (%)	Item		Frequency (%)
Sex	Male	27 (48.2%)	Place of work	Urban	14 (25%)
	Female	29 (51.8%)		Rural	42 (75%)
Age	Below 20	1 (1.8%)	Professional experience	0–2 years	9 (16.1%)
	20–30	13 (23.2%)		3–5 years	15 (26.8%)
	31–40	27 (48.2%)		6–10 years	11 (19.6%)
	41–50	10 (17.9%)		11–15 years	7 (12.5%)
	51–60	5 (8.9%)		Above 15 years	14 (25%)
Education level	A2	8 (14.3%)			
	A1	34 (60.7%)			
	A0	13 (23.2%)			
	Masters	1 (1.8%)			

The number of females (51.8%) slightly exceeds that of male participants, with the majority falling within the age group of 31–40 (48.2%). A large number of participants work in health centers in rural areas (75%). In Rwanda, an A2 nurse holds an A-level secondary school certificate (also known as associate nurse), an A1 nurse undergoes three years of university education and holds an advanced diploma (also known as registered nurse), and an A0 nurse completes four years university education, and holds a bachelor’s degree (also known as registered nurse). In this study, a substantial percentage of participants reported A1 (60.7%) as their highest level of education. Furthermore, a considerable number of participants had 3–5 years of professional experience (26.8%).

**Table 2:** Perceived behavior intention of accepting VP cases as a CPD learning approach

	Item	*N*	Median	Min	Max
Perceived ease to use	My interaction with the VP system is clear and understandable	56	5	1	5
	Interacting with the VP system does not require a lot of mental effort	56	4	1	5
	I find the VP system to be easy to use	56	5	2	5
	I find it easy to get the VP system to do what I want it to do when working with the VP case	56	4	3	5
Perceived usefulness	Using VP cases improves my skills in managing patient cases in my job	56	5	3	5
	Using VP cases for CPD will improve my productivity and learning outcomes	56	5	3	5
	Using the VP cases will enhance my effectiveness when I participate in CPD courses	56	5	3	5
	I find the VP cases to be useful for my future clinical work as a nurse	56	5	3	5
Training relevancy	In my CPD, the usage of VP cases is important	56	5	4	5
	In my CPD, the usage of VP cases is relevant	56	4	3	5
	The use of VP cases for CPD is pertinent to my various job-related tasks	56	4.5	3	5
Results demonstrability	I have no difficulty telling other healthcare workers about the importance/benefits of using VP cases for CPD	56	5	3	5
	I believe I could communicate to others the consequences of using VP cases to learn about patient cases	56	4	3	5
	The results of using the VP cases for learning patient cases are apparent to me	56	4	3	5
Perceived external control	While working with the VP system, I had control over using the VP cases	56	4	3	5
	I have the resources necessary to use the VP cases	56	5	4	5
	Given the resources, opportunities, and knowledge it takes to use the VP cases, it would be easy for me to use it	56	5	3	5
	The VP system is compatible with devices (laptops, desktop computers, tablets, and smartphones I usually use)	56	5	4	5
Behavior intention	Assuming I had access to the VP cases for my CPD I intend to use it	56	5	3	5
	Given that I get the relevant technical support for the VP cases, I predict that I will use it	56	5	3	5
	I plan to use the VP cases in the future if implemented in Rwanda to support the CPD of healthcare workers	56	5	3	5

In this study, the VP system is a web-based software, and the VP cases are simulated patient scenarios related to a disease. Table 2, presents the median, minimum, and maximum values for each statement concerning the acceptance of the VP system or VP cases. Participants used the following options for each statement: strongly disagree = 1, disagree = 2, neutral = 3, agree = 4, strongly agree = 5. The results reveal that, on each statement, the median response leans towards agree or strongly agree, with only a small number of participants strongly disagreeing, disagreeing, or expressing neutrality.

**Table 3:** The measurement constructs and their Cronbach’s Alpha

Variables	Range of scores	Mean	SD	Min	Max	Cronbach’s Alpha
Perceived ease to use	4–20	17.2	2.8	8	20	0.808
Perceived usefulness	4–20	18.4	2.1	12	20	0.892
Training relevance	3–15	13.5	1.5	10	15	0.772
Results demonstrability	3–15	13.3	1.5	10	15	0.740
Perceived external control	4–20	18.2	1.7	14	20	0.794
Behavior intention	3–15	13.6	1.5	9	15	0.773

Table 3 displays the participants’ responses regarding the constructs transformed into scales. The analysis aggregated the responses (strongly disagree = 1, disagree = 2, neutral = 3, agree = 4, strongly agree = 5) for each construct to create a continuous scale with minimum and maximum values, as illustrated in Table 3. The results suggest an overall high mean score in all the constructs used in this study to evaluate the acceptability of VP cases as the continuous professional approach. Furthermore, the average Cronbach’s Alpha exceeded 0.7 for all items, suggesting robust internal consistency [17].

**Table 4:** Correlation relationship of variables

Variables	PEU	PU	TR	SE	RD	PEC	BI
Perceived ease of use (PEU)	1						
Perceived usefulness (PU)	0.836**	1					
Training relevancy (TR)	0.689**	0.792**	1				
Results demonstrability (RD)	0.612**	0.599**	0.605**	0.429**	1		
Perceived external control (PEC)	0.683**	0.651**	0.635**	0.515**	0.699**	1	
Behavior intention (BI)	0.590**	0.567**	0.554**	0.431**	0.705**	0.714**	1

**. Correlation is significant at the 0.01 level (2-tailed)

Table 4 illustrates the correlational relationship among the constructs used in this study. The correlation results reveal that all variables correlated positively with each other (*p* < 0.001). Specifically, the results indicate that both perceived usefulness (PU) (*r* = 0.714, *p* < 0.001) and perceived ease to use (PEU) (*r* = 0.59, *p* < 0.001) are statistically significant and positively correlate with the behavior intention (BI) of using VP cases as a CPD approach.

## Discussion

This study aimed to assess the acceptability of VP cases as a CPD approach among nurses at health centers in Rwanda. The study assessed acceptability by examining the following variables: perceived ease of using the VP system, perceived usefulness of VP cases, training relevancy of VP cases, results demonstrability of using VP cases, perceived external control of VP cases, and behavior intention of accepting VP cases as a CPD approach. Generally, nurses who work at health centers and who participated in this study expressed agreement or strong agreement that VP cases can serve as an effective CPD approach.

In the technology acceptance model [8] that informed this study, they suggest that perceived ease of use and perceived usefulness are two major determinant variables that predict behavior intention of accepting a technology [8]. While perceived usefulness and perceived ease of use are key predictors of behavior intention, the technology acceptance model posits that the variables of job relevancy (referred to as training relevancy), results demonstrability, and perceived external control predict the perceived usefulness. Participants in this study agreed or strongly agreed with the statements related to these predictors and their responses showed a significant correlation with perceived usefulness. Furthermore, participants expressed consensus that the VP system applied was easy to use. Collectively, these responses paint a comprehensive picture, indicating that nurses at health centers who took part in the study have the behavioral intention of using VPs as a CPD approach. This attitude may stem from the anticipated learning benefits linked to the utilization of VP cases for enhancing their job performance [18]. Individuals intend to perform those actions with the highest product of expectancy for achieving the aspired goal [19].

Moreover, the findings in this study agree with previous studies conducted on the use of VP cases in pre-service education which have demonstrated acceptability by the students [20–22]. Nevertheless, to the best of researchers’ knowledge, this is the first study examining the behavioral intention of adopting VPs as a CPD approach among practicing nurses. According to many previous studies, the utilization of VP cases is founded on the assumption that it facilitates the exposure of students to clinical scenarios thereby enhancing their clinical reasoning abilities [23–25]. Based on this assumption, a practicing nurse has opportunities to interact with actual patients and may not necessarily require VP cases for learning. On one side, this argument holds validity, especially for a nurse or any healthcare provider working in an environment that includes both senior and junior professionals. If a such setting fosters a learning culture where senior healthcare providers actively engage in discussion with their junior counterparts regarding clinical cases, the significance of VP cases would be diminished.

However, on the other side, nurses or healthcare providers who lack the opportunity to engage in regular case discussions with their senior colleagues (as in this study setting), VP cases can serve as an approach to bridge this gap [18]. VP cases can not only feature scenarios that reflect contemporary clinical reality but also offer the opportunity for self-assessment regarding case management using feedback from experts. This is a crucial factor that can consistently enhance the quality of health service delivery, as junior healthcare professionals receive ongoing mentorship from their seniors (experts). Probably, this could explain why the nurses at health centers in this study agreed or strongly agreed that VP cases can improve their performance. In health centers in Rwanda, and likely in many Sub-Saharan African countries and other low-resource settings, nurses typically work alone in consultation rooms and make decisions that often physicians do in settings with better access to physicians and hospitals [26]. They attend to patients with various diseases and are tasked with deciding on case management or making referrals to district hospitals [26]. Most often, neither managing the case at the health center nor transferring it to the hospital provides an opportunity to receive feedback. In health centers, patients are ambulatory, and there are no mechanisms in place for nurses to assess the impact of their decisions. Similarly, when nurses transfer patients, they never receive feedback about the correctness of the motives behind their decision to transfer. In this context, learning through VP cases can offer opportunities for receiving feedback from experts, thus promoting confidence in case management [18]. As we see it, better training in the form of VP-based CPD can help to preserve scarce resources like access to physicians and time to treatment as well as prevent incidents where a patient that really should be referred to a hospital, is not transferred due to lack of experience and/or clinical reasoning skills among less experienced nurses in low-income regions.

However, the adaptation of VP cases as a CPD approach within health centers necessitates careful consideration of institutional support. “Generally speaking, people intend to perform a behavior when they evaluate it positively and when they believe important others think they should perform it” [19]. In the Technology Acceptance Model, external control is defined as the degree to which an individual believes that the organization can provide the necessary support. Previous studies on electronic devices and internet connectivity have produced varied findings: certain health centers in Rwanda are equipped with computers and have fast and stable internet connections, whereas others lack sufficient electronic devices, and face challenges with both the speed and stability of their internet [27, 28]. In this study, participants were provided with tablets and internet access, and we did not evaluate the capability of existing electronic devices and the internet infrastructure to support VP cases as a CPD approach. Nevertheless, the participants’ responses to perceived external control were positive. While some participants expressed disagreement with certain statements of this construct, the majority either agreed or strongly agreed that their institutions are capable of offering the necessary support. The positive expectation regarding the institutions’ support may stem from participants’ belief in the adequacy of existing infrastructure to facilitate the use of VP cases. Additionally, the commitment by the Rwandan government to equip all health institutions, including health centers, with fast and stable internet by the year 2024 could have influenced participants’ confidence in asserting that their institutions can offer the required support.

### Strengths and limitations of the study

The data collection for this study occurred after participants engaged in another study offering a one-week CPD program using VP cases. In this way, the nurses who responded to the questionnaire in this study had experienced VP cases for one week which enabled them to share their insights. In addition, the questionnaire was distributed electronically, enhancing anonymity and thereby providing participants with a greater sense of comfort when expressing their perceptions. On another hand, however, the criterion of including only those proficient in English might have restricted the participation of individuals with diverse perspectives. Additionally, not all participants completed the study, and the reasons for discontinuation were unknown, while the most likely explanation could be the lack of time, it is also possible that some participants found it challenging to navigate the cases or the system, or perhaps they found the approach to be uninteresting. Another limitation pertained to providing participants with borrowed tablets and internet access; probably their perspectives might have differed had they utilized locally available electronic devices and internet access.

## Conclusion and recommendations

The study findings indicate a positive inclination among nurses in Rwanda towards utilizing VP cases as a CPD approach. This acceptance holds the potential to enhance the quality of healthcare services not only in Rwandan health centers but also in other low-income countries with similar health structures. There is no reason to believe that this approach would not be effective in a similar context in middle and high-income regions of the world as well. The use of VP cases can effectively bridge the expertise gap in settings where senior staff knowledge may be limited. Constructing VP cases based on plausible contextual scenarios and guided by expert insights, can provide a consistent means to challenge nurses in health center settings and expose them to the perspectives of senior professionals.

## Data Availability

The datasets produced and examined during the present study are not publicly accessible due to data confidentiality. However, they can be obtained from the corresponding author upon request.

## Abbreviations

VP: Virtual Patient
VPs: Virtual Patients
CPD: Continuous Professional Development
VCS: Virtual Case System
SPSS: Statistical Package for Social Science
PEU: Perceived Ease of Use
PU: Perceived Usefulness
TR: Training Relevancy
RD: Results Demonstrability
PEC: Perceived External Control
BI: Behavior Intention
